# AX-Unet: A Deep Learning Framework for Image Segmentation to Assist Pancreatic Tumor Diagnosis

**DOI:** 10.3389/fonc.2022.894970

**Published:** 2022-06-02

**Authors:** Minqiang Yang, Yuhong Zhang, Haoning Chen, Wei Wang, Haixu Ni, Xinlong Chen, Zhuoheng Li, Chengsheng Mao

**Affiliations:** ^1^ School of Information Science Engineering, Lanzhou University, Lanzhou, China; ^2^ School of Statistics and Data Science, Nankai University, Tianjin, China; ^3^ School of Intelligent Systems Engineering, Sun Yat-sen University, Shenzhen, China; ^4^ Department of General Surgery, First Hospital of Lanzhou University, Lanzhou, China; ^5^ First Clinical Medical College, Lanzhou University, Lanzhou, China; ^6^ Department of Preventive Medicine, Feinberg School of Medicine, Northwestern University, Chicago, IL, United States

**Keywords:** atrous spatial pyramid pooling, boundary-aware loss function, pancreas CT, image segmentation, group convolution

## Abstract

Image segmentation plays an essential role in medical imaging analysis such as tumor boundary extraction. Recently, deep learning techniques have dramatically improved performance for image segmentation. However, an important factor preventing deep neural networks from going further is the information loss during the information propagation process. In this article, we present AX-Unet, a deep learning framework incorporating a modified atrous spatial pyramid pooling module to learn the location information and to extract multi-level contextual information to reduce information loss during downsampling. We also introduce a special group convolution operation on the feature map at each level to achieve information decoupling between channels. In addition, we propose an explicit boundary-aware loss function to tackle the blurry boundary problem. We evaluate our model on two public Pancreas-CT datasets, NIH Pancreas-CT dataset, and the pancreas part in medical segmentation decathlon (MSD) medical dataset. The experimental results validate that our model can outperform the state-of-the-art methods in pancreas CT image segmentation. By comparing the extracted feature output of our model, we find that the pancreatic region of normal people and patients with pancreatic tumors shows significant differences. This could provide a promising and reliable way to assist physicians for the screening of pancreatic tumors.

## 1 Introduction

According to the Report on Cancer from National Cancer Institute in 2021, pancreatic cancer is the third leading cause of cancer-related death in the United States (1). The identification and analysis of pancreatic region play an important role in the diagnosis of pancreatic tumors. As an important and challenging problem in medical image analysis, pancreas is one of the most challenging organs for automated segmentation, which aim to assign semantic class labels to different tomography image regions in a data-driven learning fashion. Usually, such a learning problem encounters numerous difficulties such as severe class imbalance, background clutter with confusing distractions, and variable location and geometric features. According to statistical analysis, pancreas occupies less than 0.5% fraction of entire CT volume (2), which has a visually blurry inter-class boundary with respect to other tissues.

In this article, we combine the advantages of deepLabV series, Unet, and Xception networks to present a novel deep learning framework AX-Unet for pancreas CT image segmentation to assist physicians for the screening of pancreatic tumors. The whole AX-Unet still preserves the encoder-decoder structure of Unet. In our framework, we incorporate a modified atrous spatial pyramid pooling (ASPP) module to learn the location information. The modified ASPP can also extract multi-level contextual information to reduce information loss during downsampling. We also introduce a special group convolution operation on the feature map at each level to decouple the information between channels, achieving more complete information extraction. Finally, we employ an explicit boundary-aware loss function to tackle the blurry boundary problem. The experimental results on two public datasets validated the superiority of the proposed AX-Unet model to the states-of-the-art methods.

In summary, we propose a novel deep learning framework AX-Unet for pancreas CT image segmentation. Our framework has several advantages as follows.

1. In our framework, we introduce a special group convolution, depth-wise separable convolution, to decouple the two types of information based on the assumption that inter-channel and intra-channel information are not correlated. This design can achieve better performance with even less computation than the normal convolution.
2. We restructure the ASPP module, and the extraction and fusion of multi-level global contextual features is achieved by multi-scale dilate convolution, which enables a better handling of the large scale variance of the objects without introducing additional operations. The efficacy of the restructured ASPP is validated in our ablation studies on foreground target localization.
3. We propose a loss function that can explicitly perceive the boundary of the target and combine the focal loss and generalized dice loss (GDL) to solve the problem of category imbalance. The weighted sum of the above parts is used as our final loss function, which can explicitly perceive the boundary of the target.
4. We segment a large number of external unlabeled pancreas images using our trained model. The analysis of the imagomics features of the pancreatic region shows a significant difference between patients with pancreatic tumors and normal people (*p* ≤ 0.05), which may provide a promising and reliable way to assist physicians for the screening of pancreatic tumors.

## 2 Related Work

We are developing an artificial intelligence (AI) method for medical application in this paper. In this section, we review some previous works related to our work. We first make a brief review of AI methods in medicine. Then, we focus on the research of the AI task involved in this paper (i.e., image segmentation) and review the related methods. Finally, most related to our study, we review a few representative studies that applied AI methods to medical image segmentation, especially, pancreas segmentation, and compare them with our methods.

### 2.1 Artificial Intelligence in Medicine

In recent years, with the popularization of AI technology in various fields, it has also made great progresses in the medical field. The development of AI techniques has been promoting the development of medicine, from the earliest AI methods, such as expert systems (3, 4), to more advanced statistic machine learning methods, such as support vector machine (5, 6), non-negative matrix factorization (7–9), and local classification methods (10–12). Recently, the deep learning techniques that have achieved great success in computer vision and natural language processing played an important role in the development of medicine and got great development over the past few years. Xu et al. (13) used an attention-based multilevel co-occurrence graph convolutional long short-term memory (LSTM) to enhance multilevel feature learning for action recognition. Fang et al. (14) proposed a dual-channel neural network to reduce the high noise and disturbance, which generally resides in the signal collected by wearable devices, improving the accuracy of action recognition in the process of surgical assistance and patient monitoring. Mao et al. (15–17) also employed GCN and deep generative classifiers for disease identification from chest x-rays and medication recommendation. The diagnosis of tumors based on morphological features has also found some applications, applying the morphological operators get the legion part that is possible for doctors to detect accurately where the tumor is located. Hu et al. (18) proposed an emotion-aware cognitive system. A novel undisturbed mental state assessment prototype was proposed by Giddwani et al. (19). The recent pre-trained language models are also employed for disease early prediction (20) and clinical records classification (21).

### 2.2 Image Segmentation

For the segmentation problem, many breakthroughs have been made in recent years. He et al. (22) proposed spatial pyramid pooling (SPP) to solve the fixed input size caused by the fully connected layer and proposed the parallel extraction of multi-level features of SPP layer, which makes different size inputs have output with fixed dimension. PSPNet (23) applied multi-level feature extraction to the field of semantic segmentation. In its design of pyramid pooling module, four different sizes of pooling are fused and then stitched by a bilinear interpolation and a 1 × 1 convolution. This structure is designed to aggregate contextual information from different regions, thus improving the ability to obtain global information. The DeepLabV series (24) proposed by Google later introduced ASPP in later versions, which used dilate convolution with different dilate factors to expand the receptive field without losing resolution and to fuse multi-scale context information. In addition, a 1×1 convolution and a global pooling are added in parallel. In the latest deeplabV3+ (25), the upsampling has been further refined, and better results have been achieved in boundary segmentation. In addition, in this version, Xception (26) was introduced as the backbone to perform feature extraction. This model performs channel-by-channel convolution by the assumption that the channel correlation is decoupled. Isensee et al. (27) developed nnUnet, a method that automatically configures preprocessing, network architecture, training, and post-processing for any new task, rendering state-of-the-art segmentation accessible to a broad audience by requiring neither expert knowledge nor computing resources beyond standard network training.

### 2.3 Medical Image Segmentation

Since Unet was proposed in 2015 (28), it has undergone many versions of evolution, and its performance has been continuously improved (29). Inspired by the successful application of Unet architecture and its variants to various medical image segmentations, Li et al. (30) proposed a novel hybrid densely connected UNet for liver and tumor segmentation. Yu et al. (31) used a salience transformation module repeatedly to convert the segmentation probability map for small organ segmentation. The above methods mainly use general segmentation approaches for medical image segmentation, ignoring domain-specific challenges. In the field of pancreatic segmentation, many methods have also been proposed. Farag et al. (32) used a convolutional neural network (CNN) model with dropout to conduct a classification on pixel level. Cai et al. (33) added a convolutional LSTM network to the output layer of CNN to compute the segmentation on two-dimensional (2D) slices of the pancreas. However, all of these methods merge the information between 2D slices of CT images for segmentation, which may miss some spatial information across slices. Man et al. (34) proposed a coarse-to-fine classifier on image patches and regions *via* CNN. Zhang et al. (35) proposed a new efficient SegNet network, which is composed of basic encoder, slim decoder, and efficient context block. Although these methods integrate spatial information to a certain extent, there is still room for improvement in boundary segmentation decisions. Ribalta Lorenzo et al. (36) proposed a two-step multi-modal Unet–based architecture with unsupervised pre-training and surface loss component for brain tumor segmentation which allows model to seamlessly benefit from all magnetic resonance modalities during the delineation. Shi et al. (37) presented a new semi-supervised segmentation model CoraNet based on uncertainty estimation and separate self-training strategy. The definition of uncertainty directly relies on the classification output without requiring any predefined boundary-aware assumption. Different from previous methods, our framework extracts more complete spatial and channel features, introduces multi-level and multi-scale feature extraction, and explicitly evaluates the segmentation loss of boundaries, achieving excellent results on multiple public datasets.

## 3 Methods

In this article, we propose an improved version of Unet-based backbone network, AX-Unet, incorporating a restructured ASPP module, depth-wise convolutions, and residual blocks. We also propose a hybrid loss function that is explicitly aware of the boundary.

### 3.1 Architecture

**Figure 1:**
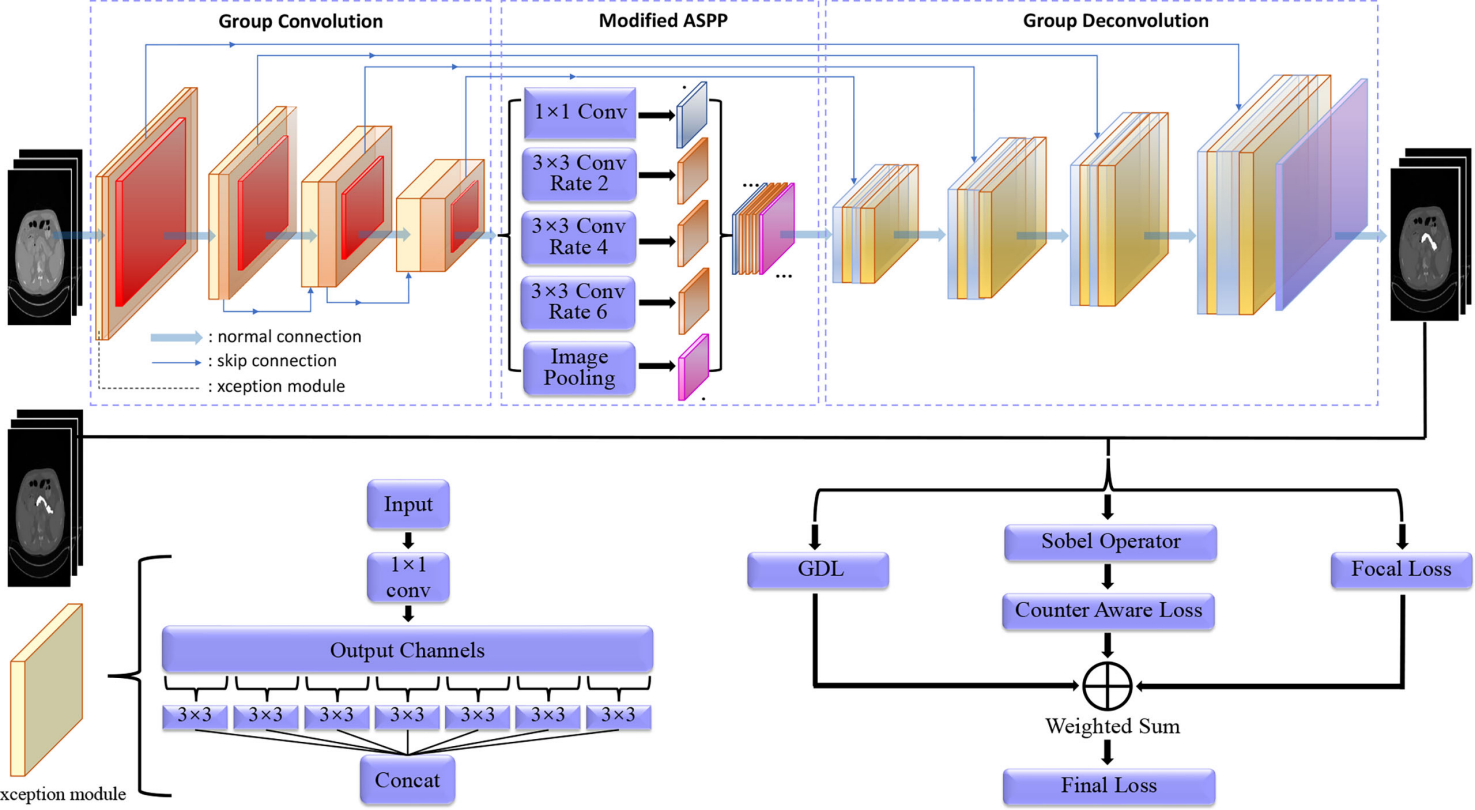
An overview of our framework. When an original image is input, first, it goes through an encoder with four downsampling blocks consisting of a group convolution (xception) and a max pooling, yielding a small feature map; then the small feature map goes through our modified ASPP module with dilated convolution to achieve multi-scale feature parallel extraction; and then the multi-scaled feature maps go through a decoder with group decovolution operations to achieve a feature map with the same shape of the original input image for pixel-wise classification. Finally, in the test process, the output feature map implies a segmentation for evaluation; in the training process, comparing the output feature map and the original input annotation to calculated the loss by the designed hybrid loss function for model training.

As shown in **Figure 1**, our model adopts a U-shaped encoder-decoder structure, which improves the basic Unet architecture in several ways. First, we replace the normal convolutions in the encoder and decoder except the first layer with group convolution, so that in the encoding process of each level, the inter-channel and intra-channel correlation information is independently extracted (38, 39). On the basis of this structure, the overlay of adjacent slices containing the foreground is used as the input of our model; in this way, we can independently extract the detailed differences between adjacent slices, which is helpful for more accurate segmentation. Therefore, in essence, the channels should be treated differently; it is better not to map them together. Second, we have added a residual structure (40) between adjacent convolution blocks, which can reduce the semantic information loss in downsampling. Third, after the encoding stage, we set up a bottleneck layer using ASPP (41), which plays an important role in extracting multi-level contextual information to reduce information loss during downsampling. By performing convolution operations on the feature maps obtained in the encoding stage in parallel with different dilated rates, the context of the image is captured at multiple scales to obtain more accurate foreground position information (42).

Because the pancreas has a small area in computed tomography images which is flexible and changeable, traditional methods may fail to find the presence of the pancreas when receiving a challenging input. The extraction of multi-level contextual semantic information is important for small and changeable target. In the decoding phase, we restore the feature maps to the original resolution of the input image layer by layer through group deconvolution (43) and reduce the number of feature maps to 2 through 1×1 convolution.

### 3.2 Depth-Wise Separable Convolution

We use a special group convolution, depth-wise separable convolution, instead of the normal convolution in the encoder. The normal convolution operation is a joint mapping of channel correlation information and spatial information in the channel (44). These two kinds of information are coupled, but the two correlations are decoupled in Inception by depth-wise convolution (45, 46). In the assumption of Inception, the two correlations are independent (47), mapping them separately can achieve better results. Because our input is in the form of numerous of slices, the independent mapping of information between channels is more reasonable. We use the extreme case of Inception, i.e., Xception in our framework, that is, the number of groups in the group convolution is equal to the number of input channels, which means inter-channel correlation and intra-channel spatial correlation are completely decoupled. The input feature map is linearly transformed channel by channel through a 1×1 convolution; the obtained feature map is fed to a number of 3×3 convolutions. Because the number of groups in our grouped convolution is equal to the number of input channels, all filters in this convolution process have a convolution kernel of 3×3, i.e., each channel of input feature map is only convolved by one kernel with size of 3×3×1. The outputs of these filters are stacked to construct the output feature map.

In terms of parameter comparison, assuming the number of input feature map is M, the number of output feature map is N, and the normal convolution kernel size is 3, the normal convolution has the number of parameters *N_n_* = 3×3×*M*×*N*, and the depth-wise separable convolution has the number of parameters from two parts, i.e., *N_g_* = *N_depth_*_−_ *_wise_* + *N_point_*_−_ *_wise_* = 3 × 3× *M* + 1 × 1 × *M* × *N*. Compared the depth-wise separable convolution with the normal convolution, the amount of parameters in our framework is reduced (48, 49), and the expressive ability of the network has been improved. In our framework, we use double convolutions for dowmsampling, in every double convolution block, we replace the first normal convolution with depth-wise separable structure Xception shown in **Figure 1**. Therefore, in each downsampling process, the convolution kernels with the same number of input channels are used to achieve information decoupling, and then, a normal convolution is used to double the number of feature maps. After calculation, if ordinary convolution is used completely, a total of 1,040,768 3×3 convolution kernels are needed in the entire downsampling process, whereas our improved structure only needs 700,544 3×3 convolution kernels.

### 3.3 ASPP Module

The pancreas images usually have blurry boundaries and are easy to be confused with surrounding soft tissues, especially, it occupies a relatively small region in a CT image with complicated background and usually less than 1.5% in a 2D image. This makes it even hard to decide whether the pancreas exists in the image. Most existing models cannot extract enough information about the position of the pancreas, which is largely related to the global context of the image. In our framework, we use an ASPP module that contains atrous convolution to improve the information extraction ability. The ASPP module is inspired by the spatial pyramid and uses multiple parallel atrous convolution layers with different sampling rates. The context in the feature map is captured at multiple scales at the same time. In the scenario where the medical image itself does not contain complex background, noise and other information, we believe that the deep and shallow features of the medical image are all important, so the fusion of different levels of features can achieve better decision-making.

As illustrated in **Figure 1**, the ASPP module that we use mainly includes the following parts:

1. A 1×1 convolutional layer and three 3×3 atrous convolutions. When the dilated rate is close to the feature map size, filters will no longer capture the global context and will be degenerated into a simple 1×1 convolution with only the filter center working. Hence, here, we scale the dilated ratio of the original module to (2, 4, 6).
2. A global average pooling layer obtains the image-level feature, and then sends it to a 1×1 convolution layer (output with 256 channels); the output is bilinearly interpolated to be the same shape with the input.
3. The four kinds of feature maps from the above two steps are concatenated together in the channel dimension and then are sent to a 1×1 convolution for fusion to obtain a new feature map with 256 channels.

To a certain extent, the ASPP module solves the defect that the traditional Unet may have in characterizing information, can better extract multi-level position information, and has stronger characterization and learning capabilities to detect and locate the pancreas. In addition, if the dilate rate is close to or even exceeds the size of the input feature map, then it will degenerate into 1×1 convolution, and a too large dilate rate will not be conducive to pixel-level output, so we use a smaller dilate rate of (2, 4, 6).

### 3.4 Hybird Loss Function

Because the region to be segmented only occupies a small part of the entire image, this imbalance of foreground and background will cause sub-optimal performance (50). In addition, the pancreas as a soft tissue, the shape is variable. On the basis of the above characteristics, we proposed a hybrid loss function to update model parameters for the pancreas study tasks where category imbalance, boundary perception, and shape perception commonly exist. Our loss function consists of the following three parts.

• Generalized dice loss:

The use of ordinary dice loss is very unfavorable for small targets. The model will be overfiting (the output is all background) because once the small target has a part pixel prediction errors, it will result in large changes in dice coefficient, which will lead to dramatic changes in gradients. Therefore, GDL imposes a weight in each segmented category so as to balance the contribution of various target areas (including background) to loss.


(1) $$Loss(GDL)\;=\;1-\frac{1}{m}\frac{2\sum_{j=1}^{m}w_{i}\sum_{i=1}^{N}y_{ij}y_{ij}^{pred}}{\sum_{j=1}^{m}w_{j}\sum_{i=1}^{N}{(}_{y}i_{j}+y_{ij}^{pred})}$$

where *w_i_* is valued by


(2) $$w_{i}=\frac{1}{{\left(\sum_{i=1}^{N}y_{ij}\right)}^{2}}$$

• Focal loss:

Focal loss is designed to solve the serious imbalance in the proportion of positive and negative samples in target detection. Focal loss is optimized on the basis of the cross-entropy loss as Equation (3), where *y* > 0 reduces the loss of easy-to-classify samples (*y^pred^* →0 or *y^pred^* →1) and pays more attention to difficult, misclassified samples (*y^pred^* around 0.5). In addition, the balance factor *α* is added to balance the uneven ratio of positive and negative samples. Here, we go to set *α* to 0.25, that is, we think negative samples are easier to distinguish.


(3) $$Focal\;Loss=\;\left\{\begin{array}{rrr} -\alpha {\left(1-y^{pred}\right)}^{\gamma }\text{log }y^{pred} & \text{for} & y\;=\;1\\ -\left(1-\alpha \right){\left(y^{pred}\right)}^{\gamma }\text{log }\left(1-y^{pred}\right) & \text{for} & y\;=\;0 \end{array}\right\}$$

• Counter-aware loss (CAL):

Pixels located at the boundary between background and foreground are so ambiguous that it is difficult to determine their labels even for experienced people. From the perspective of features, these vectors extracted from motley image pixels fall near the hyperplanes, acting as hard examples. As general networks only apply pixel-wise binary classification, target boundaries and interior pixels are processed indiscriminately using the cross-entropy loss function, so they usually predict broad outline of target objects, inferior in precision. Here, we designed a loss function based on a fixed edge extraction filter operator. The result of each iteration and the label are convolved separately. After processing, MSSS-IM (Multi-Scale-Structural Similarity Index), which measures the similarity of the image structure, is used as a loss function. This kind of explicit boundary extraction solves the problem of fuzzy boundary information and can better return the loss of boundary information.

There are many operators in edge extraction, such as Prewitt operator, Sobel operator, and Prewitt operator. They have different emphases and tendencies in boundary extraction. For example, Sobel operator detects edges according to the phenomenon of reaching extreme values at edges, which has a smoothing effect on noise. The effect of Roberts operator in detecting horizontal and vertical edges is better than that of oblique edges, and the positioning accuracy is high, but it is sensitive to noise. We choose to use the Sobel operator, which contains two sets of 3×3 matrices, which are horizontal and vertical templates, so that they can do plane convolution with our original label and segmentation output at the same time, and then, the horizontal and vertical brightness difference approximations can be obtained, respectively.

The specific two convolution operator parameters are shown in the following matrix:


$$G_{x}\;={\left[\begin{array}{lll} -1 & 0 & 1\\ -2 & 0 & 2\\ -1 & 0 & 1 \end{array}\right]}_{3\times 3}$$

$$G_{y}=\;{\left[\begin{array}{lll} 1 & 2 & 1\\ 1 & 0 & 0\\ -1 & -2 & -1 \end{array}\right]}_{3\times 3}$$

Through the calculation of convolution and gradient, we get the edge of the predicted label and the original label, respectively, and then calculate loss through the cross-entropy loss function as part of the previous loss.

Our final loss function is the weighted sum of the above three loss functions as in Equation (4).*w*_1_, *w*_2_, and *w*_3_ are tuned for different segmentation tasks. For all the pixels that are truly located in the pancreas region, we believe that the pixel values at the border are more indistinguishable, under this scene, we tune the weights of the three loss functions through grid search in range [0.2, 0.8] with step 0.2, try different combinations of weights, and finally find that, when a relatively large weight is given to CAL, the value of distance decreases significantly and dice score has also been improved to a certain extent, which proves the effectiveness of the perceptual boundary method we designed. However, when too large weight is given to the CAL, there will be many samples’ target foreground cannot be found. We think this is caused by the fact that CAL itself cannot handle the problem of extreme class imbalance of samples, so focal loss and Dice loss are still required to a certain extent. Finally, we determined through experiments that GDL, focal loss, and CAL were given 0.2, 0.2, and 0.6, respectively, based on the validation performance.


(4) $$Final\;Loss\;=\;w_{1}\times CAL+w_{2}\times Focal\;loss+w_{3}\times GDL$$

where *w*_1_, *w*_2_, and *w*_3_ represent the weights of the three loss functions.

## 4 Experiments and Results

### 4.1 Datasets

Following previous work of pancreas segmentation, two different abdominal CT datasets are used:

- As one of the largest and most authoritative Open Source Dataset in pancreas segmentation, the NIH pancreas segmentation dataset sourced from TCIA (The Cancer Imaging Archive) provides an easy and fair way for method comparisons (51). The dataset contains 82 contrast-enhanced abdominal CT volumes. The resolution of each CT scan is 512 × 512 × L, where L have a range of 181 to 466 which is the number of sampling slices along the long axis of the body. The dataset contains a total of 19,327 slices from the 82 subjects, and the slice thickness varies from 0.5 to 1.0 mm. Only the CT slices containing the pancreas are used as input to the system. We followed the standard four-fold cross-validation, where the dataset is split to four folds, each fold contains images of 20 subjects, and the proposed model was trained on 3 folds and tested on the remaining fold.
- The Medical Segmentation Decathlon (52) is a challenge to test the generalizability of machine learning algorithms when applied to 10 different semantic segmentation tasks. In addition, we use the pancreas part in modality of portal venous phase CT from Memorial Sloan Kettering Cancer Center. We used the official training-test splits where 281 subjects are in training set and 139 subjects are in test set.

### 4.2 Evaluation Metric

The performance of our approach on pancreas segmentation was evaluated in terms of dice similarity coefficient (DSC)


(5) $$DSC\left(Z,Y\right)\;=\;\frac{2\times \left|\text{Z}\cap \;Y\right|}{\left|Z\right|\;+\;\left|Y\right|}$$

where *Z* is the predicted segmentation and *Y* is the ground truth. We reported the maximum, minimum, and average values of DSC score over all testing cases in the NIH dataset and MSD dataset (52).

Moreover, we also use Jaccard coefficient, recall, and precision as auxiliary metric:


(6) $$Jaccard\left(U,V\right)\;=\;\frac{\left|U\cap \;V\right|}{\left|U\cup \;V\right|}$$

Where *U* and *V* represent the real pancreatic area and the predicted pancreatic area (pixel level), respectively.


(7) $$Precision\;=\;\frac{TP}{TP+FP}$$

(8) $$Recall\;=\;\frac{TP}{TP+FN}$$

In addition, for the metric of the segmentation problem, although Dice and others can well reflect the difference between the segmentation effect and the actual situation, its defect is insensitivity to differences in target boundaries, and the focus is mainly on the inside of the mask, while the Hausdorff distance (HD) as a measure of shape similarity, can be a good complement to Dice. In a 2D plane, HD refers to the maximum of all distances from one set to the nearest point between another set. Given two finite set of points *A* = {*α_1_*,…*α_p_*} and *B* = {*b_1_*,…*b_p_*}, the HD between them is defined as follows:


(9) H(A,B)=max{h(A,B), h(B,A)}

where h(A,B)=maxa∈Amaxb∈B∈a−b∈ , h(B,A)=maxb∈Bmaxa∈A∈b−a∈ , ║ ║ is a distance norm defined on point set A and point set B. We use the Euclidean distance representation directly.

### 4.3 Implementation Details

We implement our approach base on PaddlePaddle platform on a server equipped with V100 Tesla GPU with 32-GB memory. We use four-fold cross-validation for training and use min max normalization to scale the pixel values of the original image to [0, 1] and performed independently on the training and test sets. We found that RMS optimizer has a faster convergence speed than the Adam optimizer. Although adaptively reducing the learning rate, RMS optimizer can still get convergence on a smaller number of iterations. Thus, we used RMS as our optimizer. Our complete source code is available at Github https://github.com/zhangyuhong02/AX-Unet.git. We list our hyperparameters and system settings in **Table 1**.

**Table 1:** Hyperparameters and device parameters.

Parameter	Value
Initial learning rate	0.001
Batch size	32
Epochs	150
Optimizer	RMS
Learning rate decay	fixed size
convolution kernel size	3×3
PaddlePaddle	2.1.2+cu101
CUDA	10.1
python	3.7
GPU	*TeslaV*100 × 4
RAM	128GB

Because the method that we proposed achieves a variety of improvements in multiple levels of the network structure such as loss function, deep supervision and the form of deep supervision, we compare with the state-of-the-art methods in terms of multiple improvement direction control variables and the combined effects of each improvement structure.

**Figure 2:**
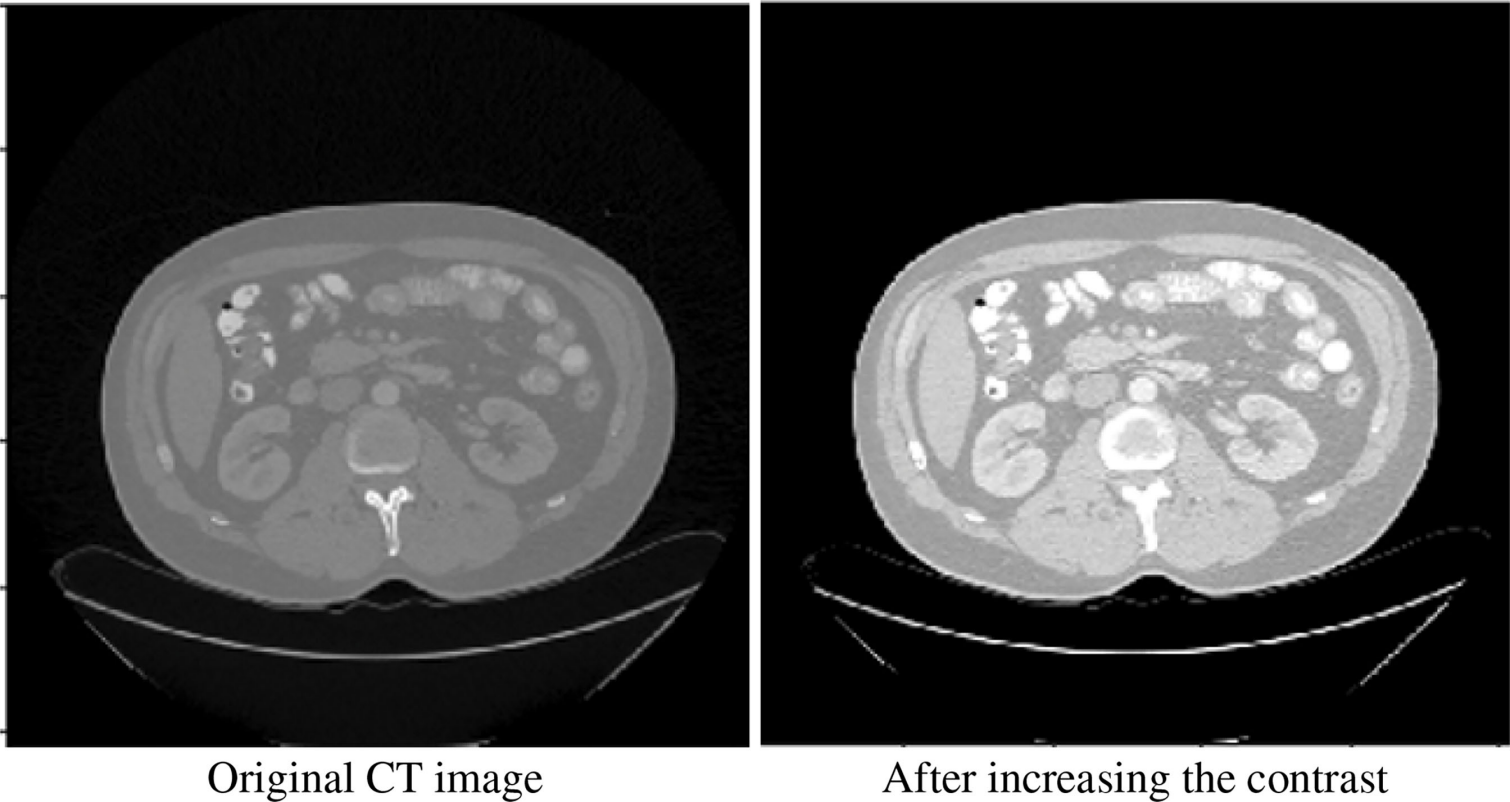
Original image and contrast-enhanced image.

We performed some basic processing on the original image. We performed 2.2 times contrast enhancement (the best performance can be obtained through hyperparameter grid search). **Figure 2** shows our comparative data enhancement effect.

### 4.4 Results

**Figure 3:**
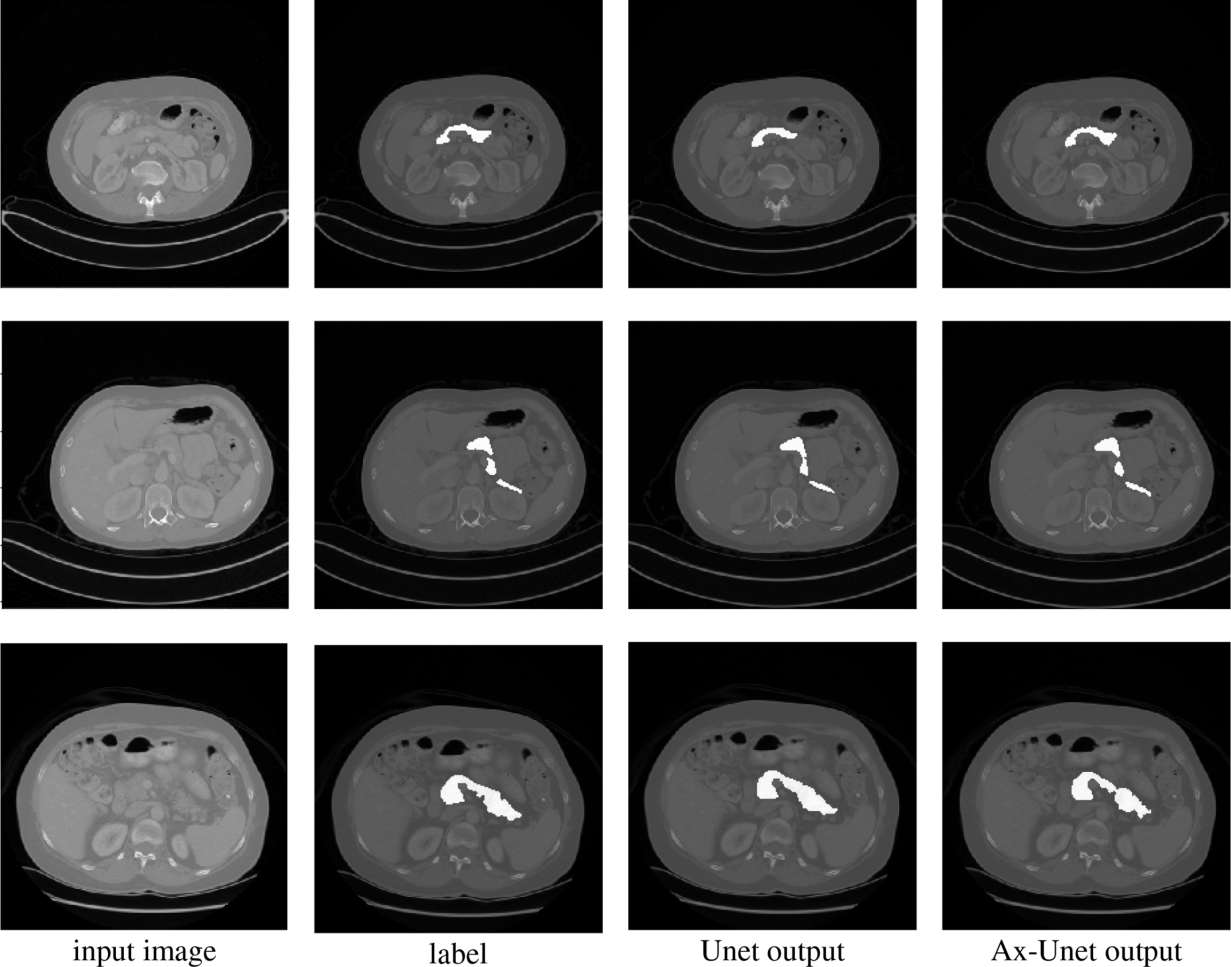
Comparison of segmentation for three examples by the baseline model (Unet) and the AX-Unet, along with the original image and ground truth. In each row, from left to right, the images correspond to the original image, ground-truth segmentation, the baseline segmentation by Unet, and segmentation by our AX-Unet model, respectively. It can be clearly observed that the proposed model has better segmentation effect of the boundary than the baseline.

In this section, we compare our proposed method with the state-of-the-art methods for image segmentation. **Table 2** shows the segmentation performance on NIH and MSD datasets in terms of DSC, Jaccard, precision, and recall. From **Table 2**, our framework can outperform the other state-of-the-art methods by a wide margin in terms of DSC, Jaccard, precision, and recall. The mean HD between out segmentation and the ground truth is 4.68, with a standard deviation 1.76. **Figure 3** shows three examples of our segmentation results. We initialized different training parameters and conducted 15 independent repeated experiments on the NIH dataset and recorded the dice score for each trained model. The mean dice score is 87.67, and the standard deviation is 3.8. We compared our results on NIH dataset with state-of-the-art methods through one sample t test, as shown in **Table 3**. From **Table 3**, our proposed method has statistically significant improvements (*p* < 0.0001) compared with other methods.

**Table 2:** The average four-fold performance on two public dataset (the performance of our method is described by *mean* ± *std*).

**Method**	**DSC (%)**	**Jaccard (%)**	**Recall (%)**	**Precision (%)**
NIH dataset				
Bottom-up (32)	70.7	57.9	71.6	74.4
Fixed-point (53)	82.4	–	–	–
3D Coarse-to-Fine (54)	84.6	–	–	–
Holistically nested (55)	81.3	68.9	–	–
RSTN (31)	84.5	–	–	–
Recurrent Contextual Learning (39)	83.3	71.8	84.5	82.8
Vnet (56)	80.1	–	–	–
Attention Unet (57)	83.1	–	–	–
DenseASPP (40)	85.4	–	–	–
(46)	84.10	72.86	85.3	83.6
Cascaded FCN (23)	85.9	75.7	85.2	87.6
AX-Unet (Ours)	**87.7 ± 3.8**	**78.2 ± 5.3**	**90.9 ± 2.2**	**92.9 ± 6.1**
MSD dataset				
Unet-64	70.7	–	–	–
Unet-16	67.1	–	–	–
Attention Unet (57)	66.0	–	–	–
MoNet (58)	74.0	68.9	–	–
nn-Unet (27)	80.0	–	–	–
AX-Unet (Ours)	**85.9 ± 5.1**	**77.9 ± 3.4**	**86.3 ± 5.1**	**93.1 ± 6.9**

**Table 3:** t-value and p-value for our method by one sample t-test.

Methods	t-value	p-value
RSTN (31)	9.2338	4.02 × 10^-7^
3D Coarse-to-Fine (54)	8.9403	8.92 × 10^-7^
DenseASPP (40)	6.5921	1.28 × 10^-5^
Cascaded FCN (23)	5.1245	0.0001

#### 4.4.1 Ablation Experiment

**Figure 4:**
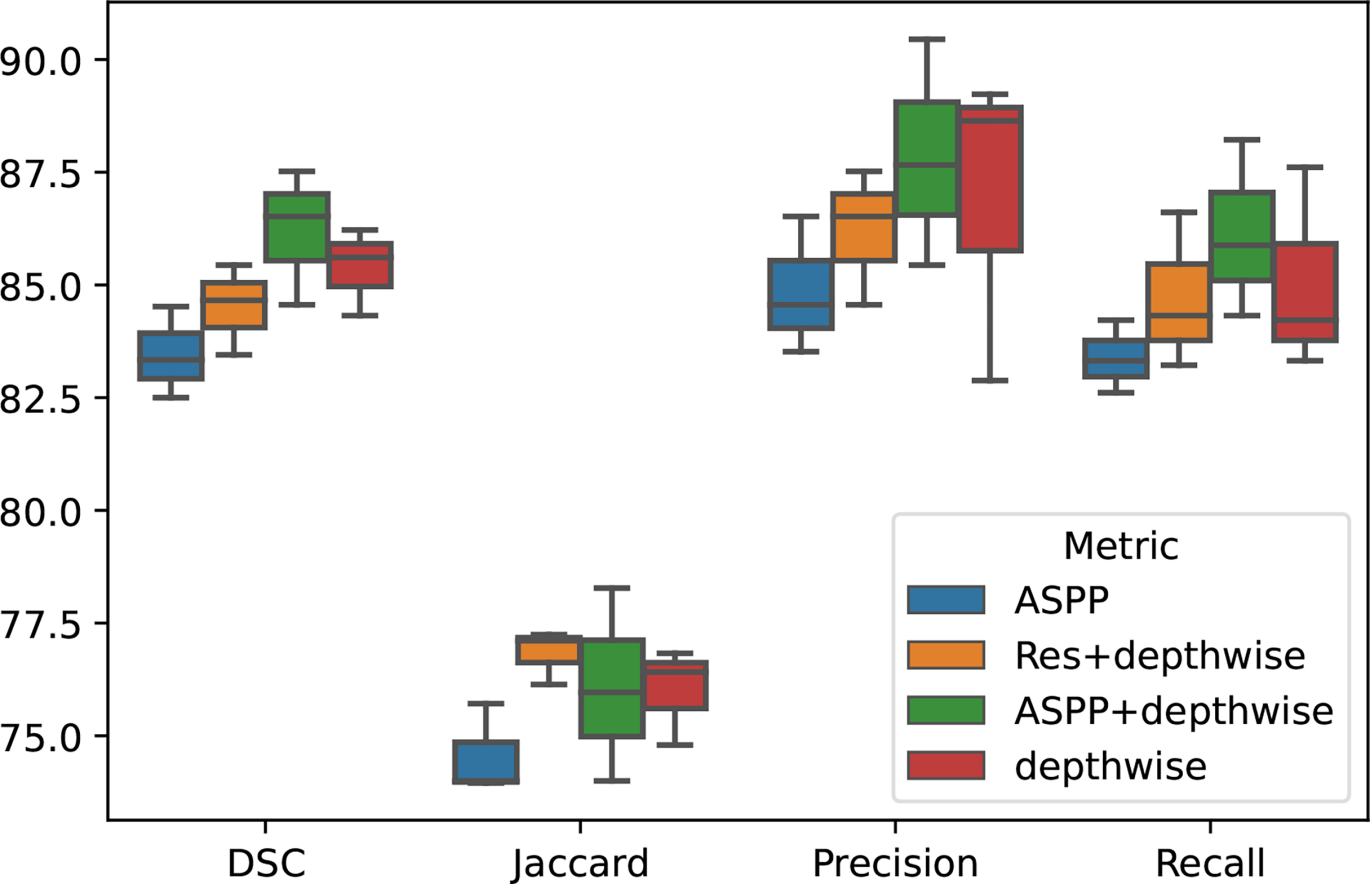
Ablation experiment on different group of module proposed in our paper.

To demonstrate the effectiveness of our group convolution and other structures, we conducted an ablation experiment to evaluate the effects of each part in our framework, residual structure, depth-separable convolution module, and ASPP module on the segmentation results. We conduct experiments using separate additional structures or different combinations of the proposed structures and perform the four-fold cross-validation on the same NIH dataset, and we repeated the experiments with different initializations for 10 times. The results are shown in **Figure 4** and **Table 4**.

**Table 4:** Results of ablation studies with different components.

Method	Jaccard (%)
Residual block	69.7 ± 8.9^**^
ASPP module (2,4,6)	76.5 ± 4.9^**^
Resuidual+ASPP(2,4,6)	76.8 ± 6.4^**^
depth-separable conv	77.4 ± 4.3^*^
Residual block+Depth-separable conv	76.7 ± 6.2^*^
Depth-separable conv+ASPP(2,4,6)	77.8 ± 3.2^*^
all	78.2 ± 5.3

The performance of different substructures is described by ***mean* ± *std***; the t-test was used for significance analysis, in which the all group containing all structures was the control group; ** indicated extremely significant difference (*p* < 0.01);* indicated significant difference (*p* < 0.05).

It can be seen that the depth-wise separable convolution achieves the greatest performance improvement when using only a single part, which validates the effectiveness depth-wise on the two correlation decoupling operations. Although the introduction of ASPP module alone did not achieve better results, the combination with depth-wise separable convolution achieved very good results. Combining all the proposed modules can achieve the best performance.

#### 4.4.2 3D Rebuilding

**Figure 5:**
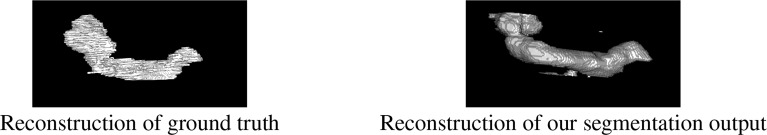
The results of 3D rebuilding. The left picture is the reconstruction of ground truth, and the right picture is the reconstruction of the segmentation output of our model.

To better demonstrate our segmentation effect, besides the segmentation results in **Figure 3**, we also show an example of the 3D rebuilding results based on our segmentation in **Figure 5**. From **Figure 5**, the rebuilding results based on our segmentation are similar with that from the ground truth, which validates the efficacy of our model.

### 4.5 Activation Map

Besides giving the segmentation results, the network can also output the activation maps of each layer, which could show a clear decision making process and give a clear medical evidence. Analyzing the activation map in the forward propagation process of the neural network can help to understand the decision making process of the model, thereby helping clinicians to achieve procedural diagnosis and more accurate treatment selection.

We extract the feature maps after each pooling in the downsampling process, take the average and maximum values of the feature maps in different levels in the channel dimension, and convert them into activation maps for visualization.

**Figure 6:**
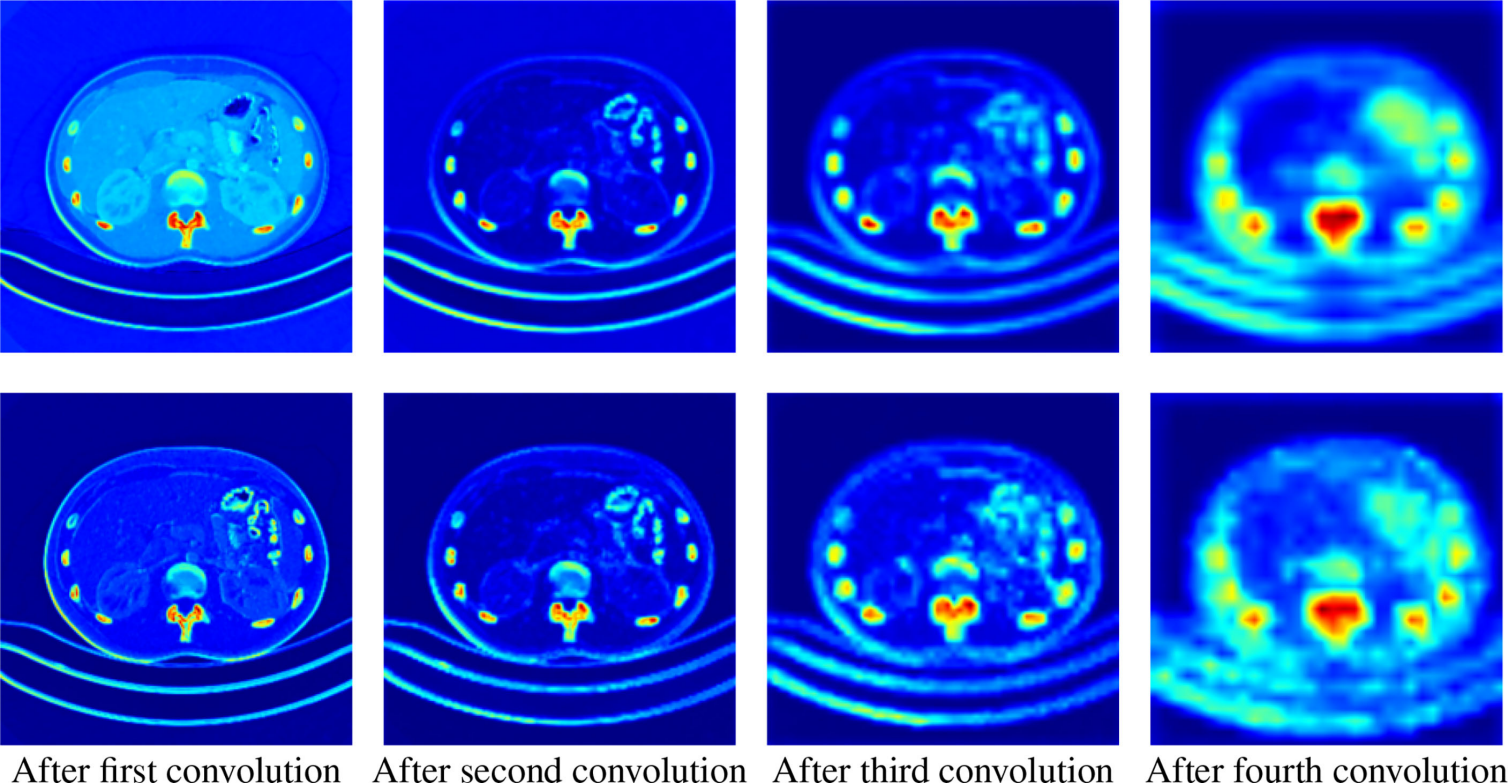
Activation maps transformed from feature maps of different levels. The upper row is the average activation maps over channels, and the lower row is the max activated maps over channels. From left to right, the activation maps are from the output of the first to the fourth downsampling block, respectively.

As shown in **Figure 6**, we extract the activate map after the pooling operations in two ways. The first row represents the activate map obtained by averaging the corresponding pixel values of each channel of the feature map of the specified level. The second row represents the activate map obtained by taking the maximum value of the corresponding pixel value of each channel. It can be clearly seen that the high-level feature maps have low resolution but strong semantics during downsampling, whereas the low-level feature maps have high resolution and rich details. This illustrates the necessity of our fusion of feature maps at different levels.

## 5 Pathological Analysis of Pancreatic Tumors With our Model

As we introduced before, the diagnosis of tumors based on morphological features has been used in brain tumors and other fields. To test the segmentation performance of our model in more complex scenarios and broaden its application scenarios, we use the proposed model to extract imagomics features for analysis. To further explore the relationship between pancreatic tumors and imagomics features and to verify the robustness of our model, we collected a large number of unlabeled data and used our pre-trained model for few-shot learning to identify pancreatic regions, followed by imagomics feature extraction and significant difference analysis.

### 5.1 Data Collection and Processing Methods

We collected pancreas image data from 49 patients from The First Hospital of Lanzhou University, which contains 31 pancreatic tumor patients and 13 normal subjects. The ages ranged from 18 to 76 years with a mean (std) of 46.8 (16.7). The CT scans have resolutions of 512 × 512 with pixels. The slice thickness is between 1.5 and 2.5 mm. The CT imaging was created using Somatom Sensation scanner with the following parameters: craniocaudal abdominal scan (120-kVp tube voltage). We manually annotated pancreas images of five individuals for the fine-tuned task and used the best performing model on the NIH Dataset as our pre-trained model. A medical student manually performed slice-by-slice segmentation of the pancreas as ground truth, and these were verified by an experienced radiologist.

### 5.2 Ethical Approval

Institutional Review Board (IRB) approval was obtained prior to the collection of the dataset. The institutional review board of the first hospital of Lanzhou university approved this study and waived the need for informed consent.

### 5.3 Transfer Learning and Feature Extraction

Through transfer learning, we fine-tuned the model trained on the public dataset on a small number of labeled samples from our dataset dataset. Then, we segmented the unlabeled data and extract 10 representative texture features from the segmentation results for pathological analysis of tumors. The features we extract are entropy (10), energy (11), homogeneity of the gray level co-occurrence matrix (glcm) (12), glcm dissimilarity (13), edge sharpness (Acu) (14), contrast (15), gray mean (59), glcm contrast (GC), glcm mean, and glcm std (60).

Contrast reflects the definition of graphics and the depth of texture, which can measure the distribution of pixel values and the amount of local changes in the image. Energy is a measure of the stability of image texture gray changes, which reflects the uniformity of image gray distribution and texture thickness. Entropy is used to measure the randomness (i.e., intensity distribution) of image texture and characterize the complexity of the image. In addition, other features are calculated based on the gray level co-occurrence matrix, which can reflect the comprehensive information of image gray level about direction, adjacent interval, change amplitude, etc. The local model of the image and the arrangement rules of the pixels are used for analysis.

In Equations (10) to (15), S, E, GH, GD, Acu, and C represent entropy, energy, homogeneity and dissimilarity of gray-level co-occurrence matrix, sharpness of image edges, entropy, and contrast, respectively, and *P_ij_* stands for the position of the current pixel.

**Figure 7:**
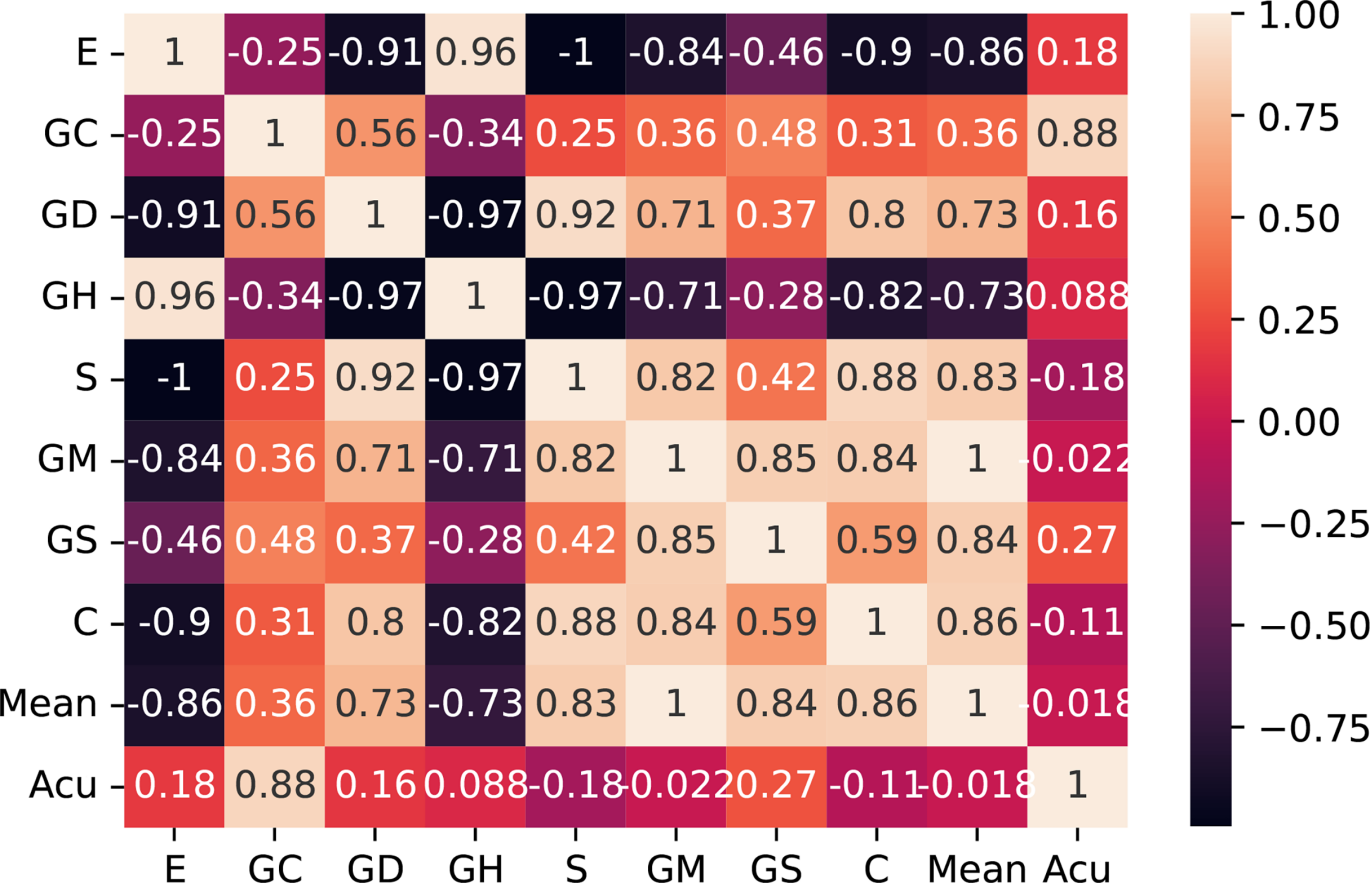
Correlation matrix with Pearson correlation coefficient of the 10 features. E, entropy; GC, gray-level co-occurrence matrix contrast; GD, gray-level co-occurrence matrix dissimilarity; GH, gray-level co-occurrence matrix homogeneity; S, entropy; GM, gray mean; GS, gray standard deviation; C, contrast; Acu, sharpness of image edges.

Then, we checked the correlation of the extracted features themselves and screened out the irrelevant features with comparison differences. After comparative analysis, we eliminated the energy and glcm dissimilarity that were highly correlated with other features. As shown in **Figure 7**, we use the Pearson correlation coefficient to measure the correlation between variables and find that energy and glcm dissimilarity are highly correlated with other features.


(10) $$S\;\;=\;\sum_{i,j=0}^{N-1}P_{i,j}\left(-lnP_{i,j}\right)$$

(11) $$E\;=\;-\sum_{i}\;\sum_{i}P_{i,j}^{2}$$

(12) $$GH\;=\;\sum_{i,j=0}^{N-1}\;\frac{P_{i,j}}{1+\;{\left(i-j\right)}^{2}}$$

(13) $$GD=\;\sum_{i,j=0}^{N-1}P_{i,j}\;\left|i-j\right|$$

(14) $$Acu\;=\;{\sum_{i}\sum_{j}\left[P_{i,j}-\mu \right]}^{2}$$

(15) $$C\;=\;\sum_{i,j=0}^{N-1}P_{i,j}\;{\left(i-j\right)}^{2}$$

### 5.4 Results and Discussion

**Figure 8:**
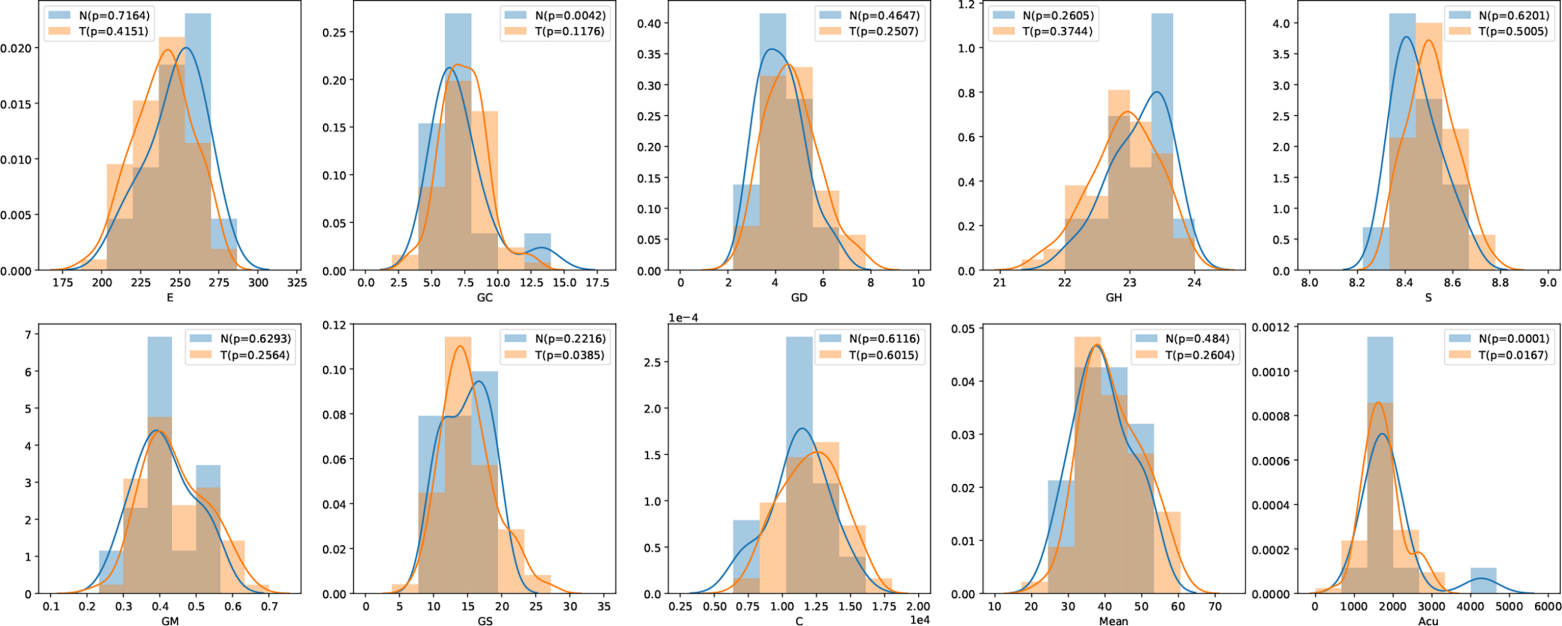
Feature distribution visualization. N represents the group of normal subjects, and T represents the group of pancreatic tumor patients. p value is the results of Shapiro–Wilk test.

**Figure 9:**
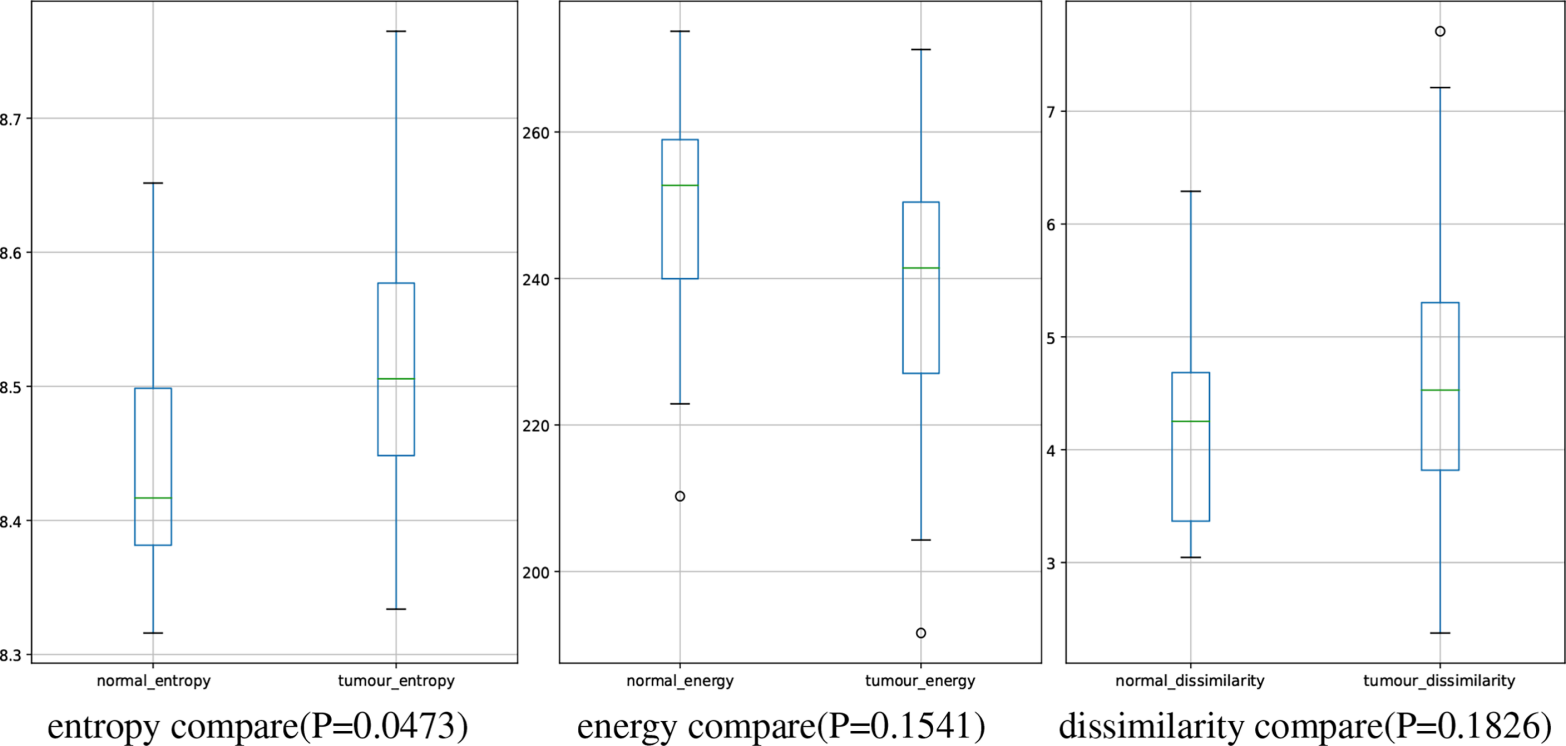
Boxplot of numerical distributions with different features.

In this study, we have 31 pancreatic tumor patients and 13 normal subjects. After the features are extracted, we use the Shapiro–Wilk test to check how likely the extracted features follow a normal distribution. Feature distribution visualization and the results of the Shapiro–Wilk test are shown in **Figure 8**. Although most of the distributions have a p-value of the Shapiro–Wilk test more than 0.05, it can be found that most of the features’ distribution is skewed to some extent, and it is safe to use a non-parametric test for significant difference analysis. We performed a Mann–Whitney U rank test to test whether a certain characteristic is significantly different between pancreatic tumor patients and normal subjects. After our calculation, it was found that the entropy extracted from the segmented images was significantly different between pancreatic tumor patients and normal people (*P* ≤ 0.05). The box plot of entropy, energy, and dissimilarity is shown in **Figure 9**. We believe that the feature entropy extracted from the output segmentation of the model is helpful for pancreas tumor diagnosis.

Entropy represents the feature of increased cellular heterogeneity during the differentiation of normal tissue into tumor tissue, which not only can reflect the difference in entropy between the two tissues on CT images but also can predict tumor recurrence and metastasis. For example, entropy can predict the pathological grade in pancreatic neuroendocrine tumors; while the entropy increases, the possibility of high-grade will increase. In addition, in related studies (61), image features of peritumoral tissue vary differently from pancreatic tumor, which may demonstrate the possibility of entropy for predicting recurrence of pancreas tumor and metastasis of small tumor from other organs.

By constructing such an interdisciplinary pancreas segmentation model, it can be applied to multiple topics in clinical research. It may be applied to the detection of small tumors and the relationship between pancreatic margins and pancreatic fibrosis and to explore the relationship between tumor or pancreatic tissue margins and important blood vessels, so as to make more reasonable treatment choices, implement the concept of precision surgery.

## 6 Conclusion

This paper proposes a novel deep learning framework AX-Unet for image segmentation for pancreas CT images. Facing the challenging scene of pancreatic segmentation, we analyzed the defects of the existing mainstream segmentation framework for medical images and proposed a more sophisticated network structure based on the encoder-decoder structure. We combine the ASPP module with multi-scale feature extraction capabilities and group convolutions that can decouple information. It can show excellent results when facing small targets that are blurred by the boundary of the pancreas and are easy to confuse the surrounding tissues. Finally, we used the proposed segmentation model to extract and analyze the radiomics features and found that there were significant differences in entropy between normal and pancreatic tumor patients, providing a promising and reliable way to assist physicians for the screening of pancreatic tumors.

## Data Availability Statement

The original contributions presented in the study are included in the article/supplementary materials. Further inquiries can be directed to the corresponding authors.

## Ethics Statement

The studies involving human participants were reviewed and approved by the Ethics Committee of the First Hospital of Lanzhou University. Written informed consent for participation was not required for this study in accordance with the national legislation and the institutional requirements. Written informed consent was not obtained from the individual(s) for the publication of any potentially identifiable images or data included in this article.

## Author Contributions

All persons who meet authorship criteria are listed as authors, and all authors certify that they have participated sufficiently in the work to take public responsibility for the content, including participation in the concept, design, analysis, writing, or revision of the manuscript.

## Funding

This work was supported in part by the National Key Research and Development Program of China (Grant No. 2019YFA0706200), in part by the National Natural Science Foundation of China (Grant No.61632014, No.61627808).

## Conflict of Interest

The authors declare that the research was conducted in the absence of any commercial or financial relationships that could be construed as a potential conflict of interest.

## Publisher’s Note

All claims expressed in this article are solely those of the authors and do not necessarily represent those of their affiliated organizations, or those of the publisher, the editors and the reviewers. Any product that may be evaluated in this article, or claim that may be made by its manufacturer, is not guaranteed or endorsed by the publisher.
